# Improved Leukemia Clearance After Adoptive Transfer of NK Cells Expressing the Bone Marrow Homing Receptor CXCR4^R334X^

**DOI:** 10.1097/HS9.0000000000000974

**Published:** 2023-11-03

**Authors:** Filip Segerberg, Mélanie Lambert, Laura Sanz-Ortega, Agneta Andersson, Richard W. Childs, Mattias Carlsten

**Affiliations:** 1 Center for Hematology and Regenerative Medicine, Department of Medicine, Huddinge, Karolinska Institutet, Stockholm, Sweden; 2 Université Sorbonne Paris Nord, INSERM, Paris, France; 3 Cellular and Molecular Therapeutics Branch, National Heart, Lung, and Blood Institute, National Institutes of Health, Bethesda, MD, USA; 4 Center for Cell Therapy and Allogeneic Stem Cell Transplantation, Karolinska Comprehensive Cancer Center, Karolinska University Hospital, Solna, Sweden

Acute myeloid leukemia (AML) is a poor-prognosis bone marrow (BM) malignancy. Standard treatment relies on chemotherapy and for selected patients hematopoietic stem cell transplantation. New treatments are highly warranted. Immunotherapy, including adoptive natural killer (NK) cell transfer, represents a new treatment modality. Here, we report that expanded human NK cells, genetically engineered to express the gain-of-function BM homing receptor CXCR4^R334X^, have superior homing capacity to AML-containing BM compartments resulting in improved treatment efficacy.

NK cells are lymphocytes with an innate ability to kill tumor cells. Studies have shown that donor NK cells can prevent AML relapse following HSCT^1^ and that adoptive NK cell transfer can induce remission in patients with relapsed/refractory AML and high-risk myelodysplastic syndromes (MDS).^2,3^ Although methods to further enhance the antitumor efficacy of NK cells are currently explored, few have focused on improving NK cell homing to the tumor.^4,5^ This is highly relevant for targeting BM–residing malignancies as infused NK cells have poor BM homing.^6^ The significance of improved BM homing was recently highlighted in a clinical trial where infusion of NK cells with high CXCR4 surface expression was associated with increased response rates in AML and MDS patients.^3^ Using mRNA transfection, it was demonstrated that NK cells expressing gain-of-function CXCR4^R334X^ have higher propensity for BM compartments in mice, but it is yet to be shown that this single modification results in better leukemia control by the NK cells.^7^

The main objective of this study was to address whether redirection of infused NK cells to BM compartments of AML-bearing mice can be utilized to augment leukemia clearance in vivo. Before exploring this approach in tumor-bearing animals, we first characterized how the CXCR4/SDF-1α axis affects homing of human NK cells to BM compartments of nontumor-bearing NOD.*Cg-Prkdc*^*scid*^*Il2rg*^*tm1Wjl*^Tg(CMV-IL3,CSF2,KITLG)1Eav/MloySzJ (NSG-SGM3) mice, as this strain is slightly different from the NSG used in previous work.^7^ Healthy donor blood NK cells were expanded as previously described^8^ followed by either mRNA transfection to transiently express CXCR4^R334X^ at high levels or CRISPR/Cas9-mediated *CXCR4* gene knock-out (KO) to diminish expression (Figure 1A; Suppl. Figure S1A; Suppl. Materials and Methods). These modifications, including CRISPR-mediated *CXCR4* KO, did not impact NK cell degranulation and more importantly target killing as assessed using a panel of AML cell lines relevant for this work (Figure 1B). Nor did these modifications affect viability (data not shown). Despite low baseline expression of *CXCR4*, an average KO efficacy of 43% at the genomic level (Figure 1C; Suppl. Figure S1B-D) resulted in a clear but nonsignificant reduction of CXCR4 surface expression (Figure 1A). More importantly, this was sufficient to significantly hinder NK cell migration to SDF-1α in vitro (Figure 1D) and BM homing in healthy NSG-SGM3 mice (Figure 1E). In contrast, CXCR4^R334X^ expression following mRNA transfection improved both in vitro migration to SDF-1α and BM homing in NSG-SGM3 mice. The increased BM homing was specific as no differences were observed in other organs (Suppl. Figure S1E).

**Figure 1.**
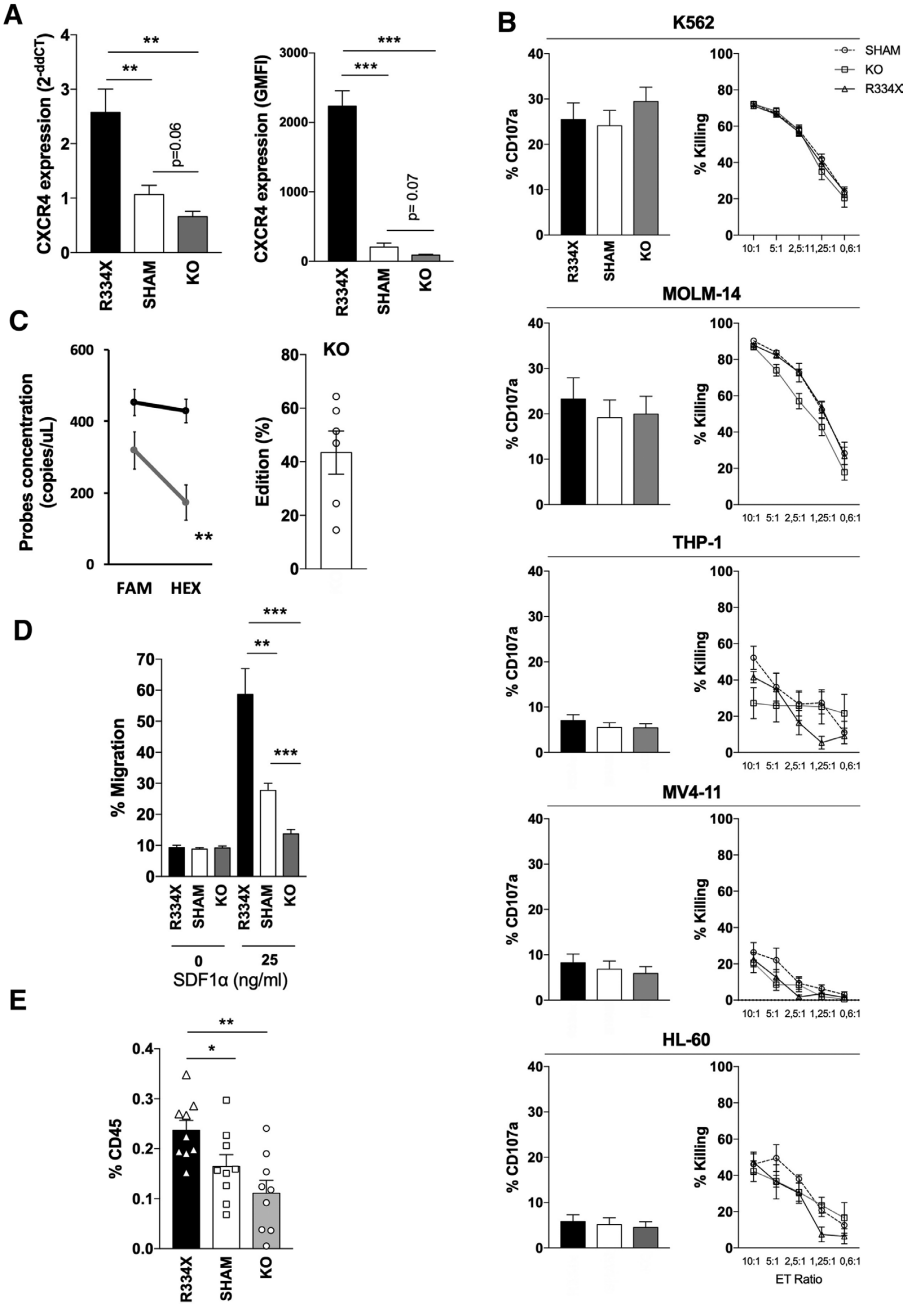
**CXCR4^R334X^ expression on expanded human NK cells improves in vitro migration to SDF-1α and in vivo bone marrow homing without impacting cytotoxicity against AML.** (A) *CXCR4* RNA transcript levels, measured by q-RT-PCR, and CXCR4 cell surface expression, measured by flow cytometry and presented as GMFI, in *CXCR4*^*R334X*^ mRNA electroporated and *CXCR4-KO* cells compared with SHAM (control) (no mRNA) electroporated cells. Both RNA transcript levels and cell surface expression of CXCR4 were evaluated 8 h post mRNA or SHAM electroporation (black and white) and 3 days post *CXCR4 KO* (gray) (n = 6). (B) Degranulation and killing assays performed 24 h post mRNA or SHAM electroporation and 4 days post *CXCR4 KO*. Degranulation was assessed by CD107a expression on the NK cell surface following coculture with different targets using flow cytometry. NK cell–mediated tumor killing was assessed on a TECAN plate reader following cocultures at different E:T ratios (n = 6). (C) Efficacy of CRISPR-Cas9-mediated *CXCR4 KO* evaluated by ddPCR analysis 3 days post electroporation. The percentage of edition was calculated from the FAM and HEX probe concentration ratio (n = 6). (D) In vitro migration toward SDF-1α was assessed 8 h post mRNA or SHAM electroporation and 3 days post *CXCR4 KO* (n = 6). (E) In vivo homing to BM compartments as determined by proportion of human NK cells defined by human CD45 and CD56 expression among total cells using flow cytometry 24 h post injection (n = 9). A total of 10 × 10^6^ NK cells were injected intravenously into nontumor-bearing NOD.*Cg-Prkdc*^*scid*^*Il2rg*^*tm1Wjl*^Tg(CMV-IL3,CSF2,KITLG)1Eav/MloySzJ (NSG-SGM3) mice (Taconic) followed by an immediate intraperitoneal injection of 100,000 IU IL-2. Unpaired t-tests were used to determine statistical significance for all subfigures above. *P < 0.05, **P < 0.01, ***P < 0.001. Where no statistical significances are shown, statistical analyses were either not performed or the results were nonsignificant. AML = acute myeloid leukemia; BM = bone marrow; *CXCR4-KO* = *CXCR4*-knock-out; ddPCR = droplet digital polymerase chain reaction; E:T = effector to target; FAX = carboxyfluorescein; GMFI = geometric mean fluorescence intensity; HEX = hexachlorofluorescein; NK = natural killer.

To address whether CXCR4^R334X^ expression on NK cells could improve their leukemia targeting capacity in BM compartments, NSG-SGM3 mice were inoculated intrabone (IB) with a high dose of MOLM-14 AML cells before receiving repeated intravenous (IV) NK cell infusions (Suppl. Materials and Methods). This model allowed us to address both leukemia reduction in the inoculated leg and spread to the noninoculated contralateral leg. A significantly reduced leukemia burden was observed in the AML-inoculated leg of mice treated with CXCR4^R334X^-expressing NK cells compared with mice receiving control or *CXCR4-KO* NK cells (Figure 2A). Notably, using CXCR4^R334X^-expressing NK cells, we also observed a significant reduction in leukemia spreading to the noninoculated leg. This finding was validated by paired comparisons between the legs, where significant differences in tumor burden were only observed in mice treated with CXCR4^R334X^-expressing NK cells (Figure 2B). Using the same model, we did not observe that CXCR4^R334X^-expressing NK cells prolonged survival (Suppl. Figure S2). We hypothesized that this discordance was caused by model-related factors. To our knowledge, there are no well-documented AML xenograft mouse models available for exploring survival post adoptive NK cell transfer. This is problematic as poor overall survival in AML is the main challenge to overcome. Indeed, many studies solemnly rely on tumor burden to measure treatment efficacy, perhaps because of difficulties to generate robust survival data. This may relate to the aggressive nature of AML cell lines in NSG-based mouse strains, where also MOLM-14 have been reported to grow rapidly.^9^ PDX models possess a possible solution to this, but can be difficult to establish, and recent evidence suggests such models favor more aggressive disease types.^10^

**Figure 2.**
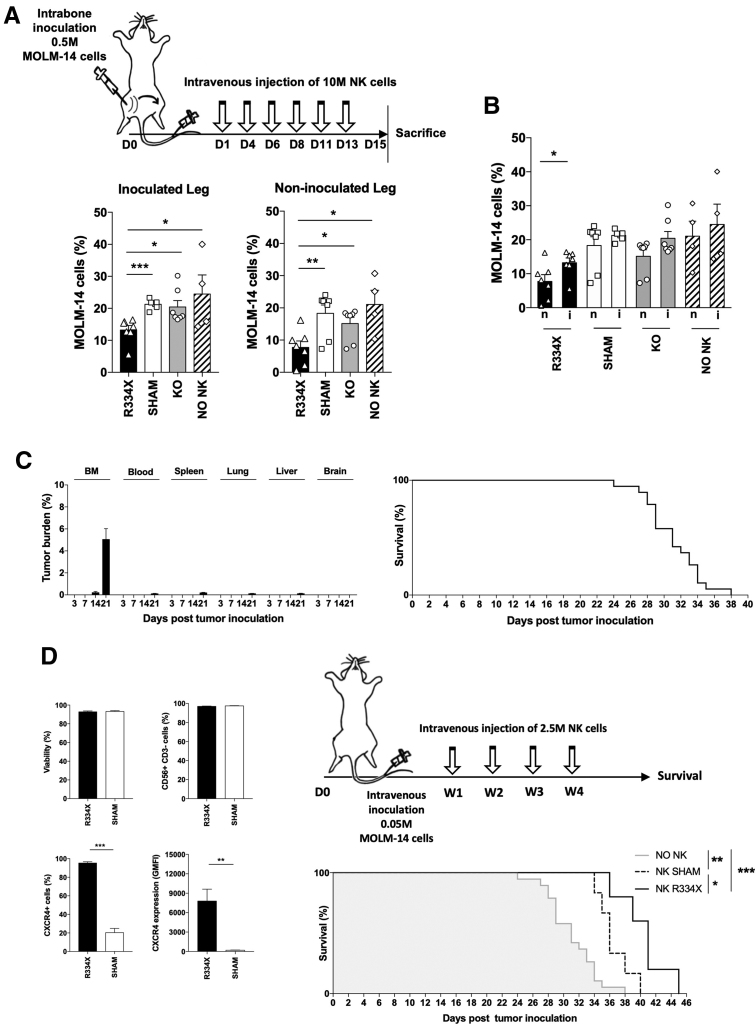
**CXCR4^R334X^-expressing NK cells induce superior leukemia clearance in the bone marrow of mice and mediate a survival benefit compared with control NK cells.** (A) The illustration outlines the experimental protocol. NSG-SGM3 mice were inoculated with 0.5 × 10^6^ GFP/Luciferase-transduced MOLM-14 cells intrabone (right hind leg) followed by intravenous injections of 10 × 10^6^ NK cells every third day for 6 cycles starting 1 day after tumor inoculation for the treatment groups; 100,000 IU IL-2 were injected intraperitoneally at the time of, and 12 h after, each NK cell injection. The bar graphs illustrate percentage of MOLM-14 cells in the BM compartments from the tumor inoculated and nontumor inoculated contralateral leg at the day of sacrifice. MOLM-14 cells were identified by human CD45 and GFP expression (n = 4–7). Unpaired t-tests were used to determine statistical significance in this subfigure. (B) The bar graphs illustrate percentage of MOLM-14 cells in the BM compartments from the nontumor inoculated (n) and the tumor inoculated (i) leg at the day of sacrifice. MOLM-14 cells were identified by human CD45 and GFP expression and assessed by flow cytometry (n = 4–7). Paired t-tests were used to determine statistical significance in this subfigure. (C) NSG-SGM3 mice were intravenously inoculated with 0.05 × 10^6^ MOLM-14 cells and either sacrificed at indicated time points for organ harvest to determine the in vivo growth pattern or kept for survival. The left graph display percentage of MOLM-14 cells, illustrated with bar graphs, in the listed organs at the specified time points after tumor inoculation for the harvested mice (n = 3/time point). MOLM-14 cells were identified by human HLA I and GFP expression assessed by flow cytometry. The right graph displays a Kaplan-Meier curve on survival (n = 19). (D) The illustration to the upper right outlines the experimental protocol. Mice within the treatment groups received intravenous injections of 2.5 × 10^6^ NK cells once per week for 4 cycles starting 3 days post tumor inoculation; 100,000 IU IL-2 were injected intraperitoneally at the time of, and 24, respectively, 96 h after, each NK cell injection. The bar graphs to the left display a fraction of SHAM and R334X electroporated cells that were saved and stained 6–8 h after each electroporation every week for assessment of viability, purity, and CXCR4 expression (denoted with both percentage and GMFI) among the cells that were intravenously injected into the mice (n = 8). The lower graph to the right display Kaplan-Meier curves on the survival of mice treated with the denoted NK cell preparations from donor 1. The median survival of untreated mice was used as a cutoff to discriminate events of early death in this analysis (n = 5–6 for treated mice, 19 for untreated mice). Unpaired t-tests and log-rank (Mantel-Cox) tests were used to determine statistical significance in this subfigure. *P < 0.05, **P < 0.001. Where no statistical significances are shown, statistical analyses were either not performed or the results were nonsignificant. FAX = carboxyfluorescein; GMFI = geometric mean fluorescent intensity; HEX = hexachlorofluorescein; NK = natural killer.

Despite the lack of well-established models, we believe survival is important to report when exploring new therapeutics. The IB model was valuable for understanding that redirection of NK cells significantly can reduce leukemia burden in the BM. However, inoculating a substantial amount of AML cells into a single anatomical compartment poorly mimics AML development and may therefore be suboptimal for assessing survival. We speculated that a reduced initial tumor load and change of administration pathway could render a slightly less aggressive model that more closely resembled disease progression in humans. Therefore, we established a model in which NSG-SGM3 mice were IV inoculated with 10-fold less MOLM-14 cells compared with the IB model, resulting in exclusive leukemia engraftment in the BM with detection of extramedullary disease after 14–21 days (Figure 2C; Suppl. Figure S3A; Suppl. Materials and Methods). Compared with the IB model, this model was less aggressive as untreated mice lived longer (median 21 versus 31 days, respectively). As NK cell cytotoxicity is donor dependent, we next evaluated survival of IV inoculated mice treated with control NK cells from 2 independent donors and identified one that prolonged survival in a dose-dependent manner (Suppl. Figure S3B,C). Using this donor, we investigated whether NK cells transiently expressing high CXCR4^R334X^ levels could further prolong survival compared with control NK cells. Although the majority of mice responded with prolonged survival, events of early death (defined as mice not surviving longer than the median of untreated mice) were observed in both treatment groups (Suppl. Figure S3D; Suppl. Materials and Methods). These early deaths were likely a consequence of inoculating a limited number of AML cells in this model, reflecting the natural trade-off between rendering a less aggressive phenotype and simultaneously introducing larger technical variance resulting in death of untreated mice spread out over a 14-day period starting at day 24 (Figure 2C). By focusing on mice that benefitted from treatment, for which we better can address the therapeutic potential of the modification, a significantly prolonged survival was observed for AML-xenografted mice treated with CXCR4^R334X^-expressing NK cells compared with control NK cells (Figure 2D).

Although these data demonstrate the therapeutic potential of CXCR4^R334X^ expressing NK cells in an AML context, we recognize that survival in this preclinical in vivo model likely can be further improved. We speculate that the transient nature of CXCR4^R334X^-expression following mRNA transfection (maximum 36–48 h^7^) could be a limiting factor but also acknowledge that a more permanent expression may not automatically lead to significant improvements in survival. As an example of the latter, Biondi et al recently showed that although anti-CD33 CAR equipped cytokine-induced killer (CIK) cells permanently expressing CXCR4^R334X^ displayed enhanced retainment in the BM, these cells had inferior ability to reduce leukemia burden in vivo and prolong survival of KG-1 inoculated mice compared with anti-CD33 CAR CIK cells engineered to express CXCR4^WT^.^11^ The authors postulated that this may be due to the fact that mutant CXCR4 receptors have been shown to interfere with T-cell receptor signaling, disrupting the immunological synapse.^12^ Furthermore, they also revealed that CXCR4^R334X^ expression decreases the ability of CAR-expressing CIK cells to bind target cells as assessed per in vitro conjugate stability assays.^11^ It should, however, be noted that these findings were made using CIK cells, a preparation primarily consisting of T cells, and that the consequences of introducing *CXCR4*^*R334X*^ in NK cells may differ. Indeed, Ng et al recently showed that anti-CD38 CAR NK cells transfected to transiently express CXCR4^R334X^ could prolong survival of multiple myeloma–bearing mice compared with controls.^13^ This indicates that the use of transient versus permanent expression may depend on which cell type that is to be used and what disease that is to be targeted. Furthermore, until we have long-lived NK cells available for adoptive infusion, transient CXCR4^R334X^ expression may be a perfectly adequate option given that multiple infusions, as used in this report, are commonly used in clinical trials.^14–16^ The mRNA approach may also have benefits over permanent expression from a safety perspective.

In summary, this letter presents data on an optimized xenograft AML mouse model to address survival following adoptive NK cell therapy and establish proof-of-concept for that NK cell–mediated rejection of AML in vivo can be augmented by exclusively redirecting their homing to the BM via transient CXCR4^R334X^ expression. Combining transient or more stable CXCR4^R334X^ expression with novel chimeric antigen receptors^17,18^ or bi- or trispecific killer engagers^19^ have the potential to further advance NK cell immunotherapy for myeloid leukemia. Future studies are warranted to ultimately define its clinical potential.

## ACKNOWLEDGMENTS

We would like to thank Iyadh Douagi, Belinda Pannagel, Mahin Nikougoftar Zarif, Francesca Grasso and Petter Woll for being in charge of the flow facility at HERM, KI. Further, the authors would like to thank Moustapha Hassan, Kristian Königsson and all staff at the animal facility PKL at KI Campus Flemingsberg. Lastly, the authors would like to thank Dr. Emily Levy for her work, including published papers and her PhD thesis, on the topic of NK cell homing and Dr. Sidinh Luc for critical input on the article.

## AUTHOR CONTRIBUTIONS

FS, ML, and MC conceptualized and designed the study. FS, ML, LSO, and AA performed experiments and collected data. FS, ML, LSO, and MC analyzed and interpreted data. FS, ML, and MC wrote the manuscript. RC provided scientific input. All authors have critically reviewed the manuscript. MC provided the financial support.

## DATA AVAILABILITY

Data included in this letter are original and can be shared upon a reasonable request to the corresponding author, Mattias Carlsten (mattias.carlsten@ki.se).

## DISCLOSURES

The authors have no conflicts of interest to disclose.

## SOURCES OF FUNDING

This work was supported by funding from Cancerfonden (MC), Swedish Research Council (MC), Stiftelsen Clas Groschinskys Minnesfond (MC), Dr. Åke Olssons stiftelse för forskning inom haematologi (MC), the Clinical Scientist Training Programme at KI (FS).

## Supplementary Material
