# Supplementary material for: Improved Leukemia Clearance After Adoptive Transfer of NK Cells Expressing the Bone Marrow Homing Receptor CXCR4R334X
Source: Hemasphere. 2023 Nov 3;7(11):e974. doi: 10.1097/HS9.0000000000000974 (PMC10627636; doi:10.1097/HS9.0000000000000974)
Supplement: Supplementary file 1 [file hs9-7-e974-s001.docx]

**SDC, Figure 1**

**
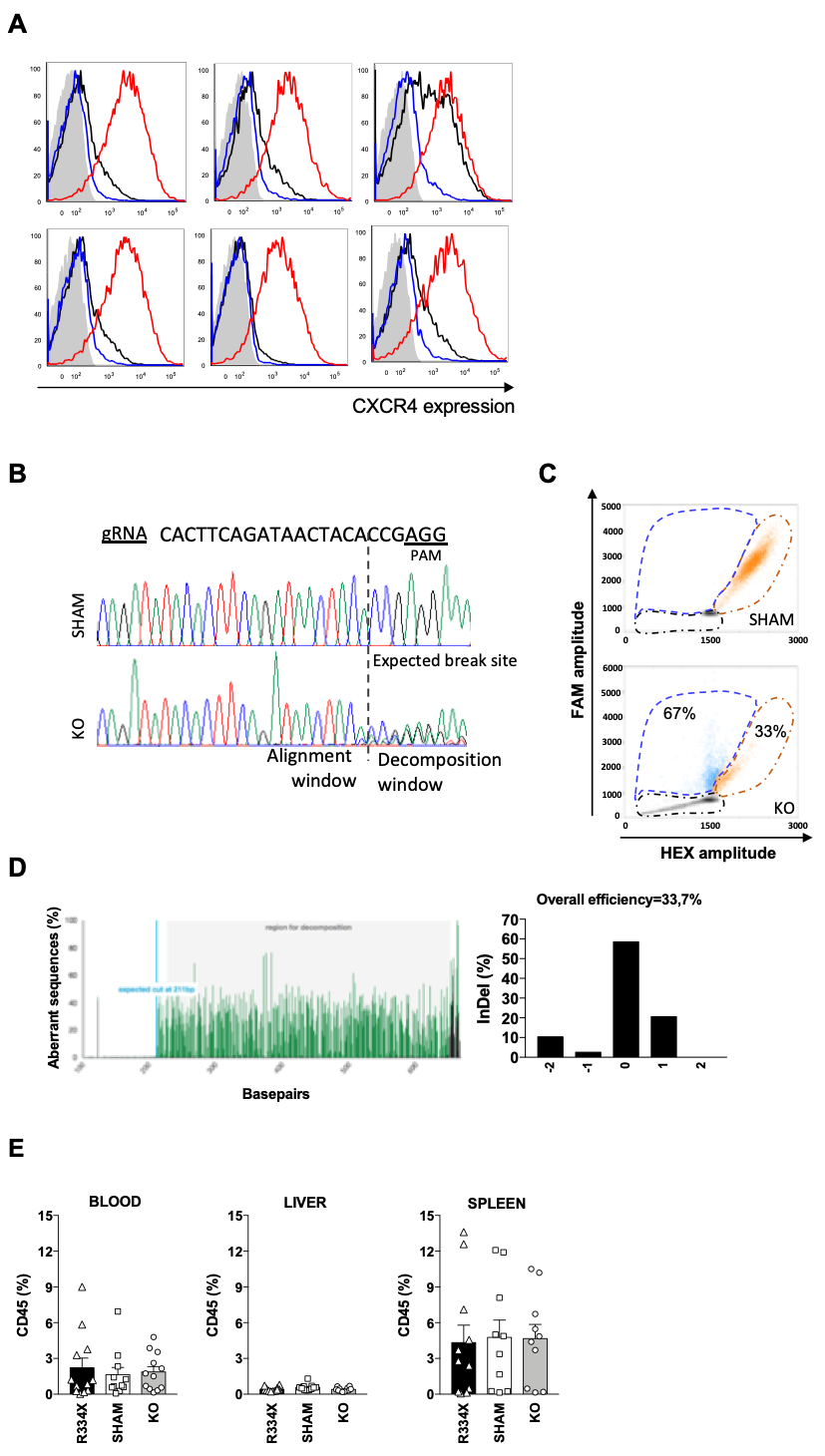
**

**Supplemental Figure 1. CRISPR/Cas9-mediated knock-out of *CXCR4* reduces CXCR4 expression by expanded human NK cells.** A) Representative histograms of CXCR4 cell surface expression quantified by flow cytometry on NK cells from 6 healthy donors. R334X (red), SHAM (black) and KO (blue). B) Illustrated are the *CXCR4* genomic edition, the guide RNA sequence used for both ddPCR and Sanger sequencing, as well as the expected cut site. C) ddPCR analysis of the *CXCR4* genomic edition in a representative donor 3 days post *CXCR4* KO, evaluated by ddPCR analysis. D) TIDE analysis expressing the percentage of potential insertion and deletion and their localization from the expected cut site, of the *CXCR4* genomic edition in a representative donor 3 days post-electroporation. E) *In vivo* NK cell distribution in blood, spleen and liver 24 hours after injection into non-AML-bearing NSG-SGM3 mice. NK cells were identified by expression of human CD45 and CD56 and quantified by flow cytometry (n = 10-12). Where no statistical significances are shown, statistical analyses were either not performed or the results were non-significant.
