# Supplementary material for: Improved Leukemia Clearance After Adoptive Transfer of NK Cells Expressing the Bone Marrow Homing Receptor CXCR4R334X
Source: Hemasphere. 2023 Nov 3;7(11):e974. doi: 10.1097/HS9.0000000000000974 (PMC10627636; doi:10.1097/HS9.0000000000000974)
Supplement: Supplementary file 2 [file hs9-7-e974-s002.docx]

**Material and methods**

*In vitro experiments*

NK cells were isolated from peripheral blood of healthy donors (Ethical approval 2006/229-31/3) and expanded as previously described.^1^ A 4D-Nucleofector^TM^ System (Lonza) or a Maxcyte GT instrument (MaxCyte) was used to introduce 4 μg mRNA (TriLink Biotechnologies)/10^6^ expanded NK cells using the CM137 and NK2 program respectively. CXCR4 gene knock-out (CXCR4-KO) NK cells were generated and KO efficacy evaluated according to previously established protocols.^2^ K562 cells and the AML cell lines MOLM-14, THP-1, HL-60 and MV4-11 (ATCC) were all cultivated in RPMI 1640 media (Gibco) supplemented with 10% fetal bovine serum (Gibco). Degranulation assays were performed by co-culturing NK cells and tumor cells at a 1:1 ratio for 1 hour. Flow cytometry staining was carried out as previously described^3^ and cells were acquired on a LSR II Fortessa or a FACSymphony A5 cell analyzer instrument (BD). NK cell-mediated tumor killing was assessed using a Calcein-AM-based assay as described elsewhere.^4^ Migration assays^3^ were performed 3 days post KO and 8 hours from mRNA transfection. Both assays were quantified on an Infinite® 200 PRO microplate spectrometer (Tecan).

*Mouse experiments*

Animal experiments were performed under ethical approval (ID1533). For BM homing studies*,* 10x10^6^ NK cells were injected intravenously into non-tumor-bearing NOD.*Cg-Prkdc^scid^Il2rg^tm1Wjl^*Tg(CMV-IL3,CSF2,KITLG)1Eav/MloySzJ (NSG-SGM3) mice (Taconic) followed by an immediate intraperitoneal injection of 100,000 IU IL-2. To evaluate the *in vivo* anti-leukemic potential of the NK cells, NSG-SGM3 mice were inoculated with 0.5x10^6^ GFP/Luciferase-transduced MOLM-14 cells intrabone (right hind leg) or 0,05x10^6^ GFP/Luciferase-transduced MOLM-14 cells intravenously, followed by intravenous injections of 10x10^6^ NK cells every third day for six cycles starting one day after tumor inoculation or 2.5x10^6^ NK cells once per week for 4 consecutive weeks starting 3 days after tumor inoculation. 100,000 IU IL-2 were injected intraperitoneally at the time of, and 12 hours after, each NK cell injection or at the time of, and 24 respectively 96 hours after, each NK cell injection. Health assessments of the mice were conducted daily and the mice were sacrificed when they reached the pre-defined criteria. In Figure 2D, The median survival of untreated mice was used as a cut-off to discriminate events of early death in this analysis, which corresponded to when an NK cell treatment effect could be detected (supplemental Figure 3B).

*Statistical Analysis*

Data were analyzed using paired t-tests for paired analyses, unpaired t-tests for unpaired analyses and log-rank (Mantel-Cox) test for survival analyses.

**References for the supplemental information**

1 Berg M, Lundqvist A, McCoy P, et al*.* Clinical-grade ex vivo-expanded human natural killer cells up-regulate activating receptors and death receptor ligands and have enhanced cytolytic activity against tumor cells. *Cytotherapy.* 2009;11(3): 341–55.

2 Lambert M, Leijonhufvud C, Segerberg F, Melenhorst JJ, Carlsten M. CRISPR/Cas9-Based Gene Engineering of Human Natural Killer Cells: Protocols for Knockout and Readouts to Evaluate Their Efficacy. In: *Methods in Molecular Biology*. Humana Press Inc., 2020, pp 213–239.

3 Levy E, Reger R, Segerberg F, et al*.* Enhanced Bone Marrow Homing of Natural Killer Cells Following mRNA Transfection with Gain-of-Function Variant CXCR4(R334X). *Front Immunol.* 2019;10:1262.

4 Segerberg F, Lundtoft C, Reid S, et al*.* Autoantibodies to Killer Cell Immunoglobulin-Like Receptors in Patients With Systemic Lupus Erythematosus Induce Natural Killer Cell Hyporesponsiveness. *Front Immunol.* 2019;10:2164.
