# Supplementary material for: Improved Leukemia Clearance After Adoptive Transfer of NK Cells Expressing the Bone Marrow Homing Receptor CXCR4R334X
Source: Hemasphere. 2023 Nov 3;7(11):e974. doi: 10.1097/HS9.0000000000000974 (PMC10627636; doi:10.1097/HS9.0000000000000974)
Supplement: Supplementary file 3 [file hs9-7-e974-s003.docx]

**SDC, Figure 2**


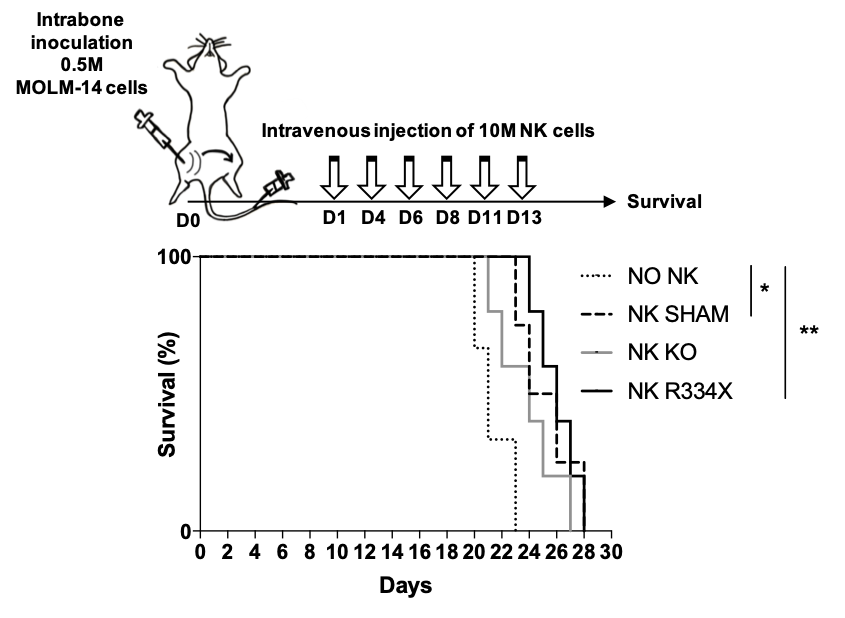


**Supplemental Figure 2. Survival after intrabone inoculation with a high dose of MOLM-14 cells illustrates the aggressiveness of AML cells in NSG-SGM3 mice.** The illustration outlines the experimental protocol. The graphs display Kaplan-Meier curves on the survival of mice treated with the denoted NK cell preparations (n = 3-5/group). Log-rank (Mantel-Cox) tests were used to determine statistical significance. *p < 0.05, **p < 0.01.Where no statistical significances are shown, results were non-significant.
