# Supplementary material for: Improved Leukemia Clearance After Adoptive Transfer of NK Cells Expressing the Bone Marrow Homing Receptor CXCR4R334X
Source: Hemasphere. 2023 Nov 3;7(11):e974. doi: 10.1097/HS9.0000000000000974 (PMC10627636; doi:10.1097/HS9.0000000000000974)
Supplement: Supplementary file 4 [file hs9-7-e974-s004.docx]

**SDC, Figure 3**

**Supplemental Figure 3. Characterization of AML disease progression in NSG-SGM3 mice after intravenous injection for optimal donor selection and survival analysis.** A) Percentage of MOLM-14 cells, illustrated with bar graphs, in the listed organs at the specified time points post tumor inoculation for the harvested mice (n = 3/time point). MOLM-14 cells were identified by human HLA I and GFP expression assessed by flow cytometry. B) Kaplan-Meier curves on the survival of mice treated with the denoted NK cells derived from two different healthy donors (n = 7-8 for treated mice, 19 for untreated mice). C) Kaplan-Meier curves on the survival of mice treated with the denoted NK cell doses from NK donor 1 (n = 8 for mice treated with NK 2.5M once/week, 3 for mice treated with NK 5M twice/week). D) Kaplan-Meier curves on the survival of mice treated with the denoted NK cell preparations from donor 1 (n = 8 for treated mice, 19 for untreated mice). Log-rank (Mantel-Cox) tests were used to determine statistical significance. *p < 0.05, **p < 0.01. Where no statistical significances are shown, statistical analyses were either not performed or the results were non-significant.
